# Improvements in the biochemical responses and Pb and Ni phytoremediation of lavender (*Lavandula angustifolia* L.) plants through *Funneliformis mosseae* inoculation

**DOI:** 10.1186/s12870-023-04265-0

**Published:** 2023-05-13

**Authors:** Farzad Rasouli, Mohammad Bagher Hassanpouraghdam, Yaghoub Pirsarandib, Mohammad Ali Aazami, Mohammad Asadi, Sezai Ercisli, Lamia Vojodi Mehrabani, Ivana Puglisi, Andrea Baglieri

**Affiliations:** 1 Department of Horticultural Science, Faculty of Agriculture, University of Maragheh, Maragheh, 55181-83111 Iran; 2 Department of Plant Production and Genetics, Faculty of Agriculture, University of Maragheh, Maragheh, 55181-83111 Iran; 3 Department of Horticulture, Faculty of Agriculture, Ataturk University, 25240 Erzurum, Türkiye & HGF Agro, Ata Teknokent, Erzurum, TR-25240 Turkey; 4 Department of Agronomy and Plant Breeding, Azarbaijan Shahid Madani University, Tabriz, Iran; 5 Department of Agriculture, Food and Environment (Di3A), University of Catania, 95123 Catania, Italy

**Keywords:** Essential oil constituents, Symbiosis, Heavy metals, FRAP, DPPH

## Abstract

**Background:**

Heavy metals (HMs) phytoremediation is a well-recognized protocol to remove toxic elements from the soil. As known, arbuscular mycorrhizal fungi (AMF) enhance the plants' growth responses. The idea of the present study was to assay the response of lavender plants to HMs stress under AMF inoculation. We hypothesized that mycorrhiza will enhance the phytoremediation and simultaneously reduce the harmful effects of heavy HMs. So, lavender (*Lavandula angustifolia* L.) plants were inoculated with AMF (0 and 5 g Kg^−1^ soil) under Pb [150 and 225 mg kg^−1^ soil from Pb (NO_3_)_2_] and Ni [220 and 330 mg kg^−1^ soil from Ni (NO_3_)_2_] pollution, in the greenhouse conditions. The control treatment was plants not treated with AMF and HMs. Doing this, the root colonization, HMs uptake, enzymatic and non-enzymatic antioxidants pool, MDA, proline, total phenolics (TPC), flavonoids (TFC), anthocyanins, and essential oil (EO) components were evaluated.

**Results:**

According to the findings, the AMF inoculation enhanced shoot and root Pb and Ni content, antioxidant enzymes activity, the total antioxidant activity by DPPH and FRAP methods, TPC, TFC, anthocyanins, and H_2_O_2_ content in the lavender plants subjected to Pb and Ni stress. Moreover, the highest (28.91%) and the least (15.81%) percentages of borneol were identified in the lavender plants subjected to AMF under 150 mg kg^−1^ of Pb and the control plants without AMF application, respectively. Furthermore, the top 1,8-cineole (12.75%) content was recorded in AMF-inoculated plants.

**Conclusions:**

The overall results verify that AMF inoculation can be a reliable methodology to enhance the phytoremediation of Pb and Ni by lavender plants while maintaining reliable growth potential. The treatments improved the main EO constituents content, especially under moderate HMs stress conditions. With more detailed studies, the results will be advisable for the extension section for the phytoremediation of polluted soils.

## Introduction

To maintain acceptable health indices; medicinal plants have been considered valuable alternatives to replace several chemical drugs and antibiotics [1]. Therefore, medicinal plants have been increasingly utilized to extract and formulate their unique secondary metabolites. Several medicinal plants' bioactive constituents are commonly used in various pharmaceutical, cosmetic, and food industries [2].

*Lavandula angustifolia* L. is an aromatic crop from Lamiaceae with a recognized antibacterial, antifungal, antioxidant, anti-inflammatory, and antiviral activities [3]. Additionally, lavender preparations are in use in patients with anxiety, sleep disorders, headaches, seizures, and depression. Linalool, 1, 8-cineole, linalyl acetate, ocimene, terpinene, and camphor are the chief bioactive components of lavender essential oil (EO) [4]. The sedative properties of lavender EO are verified with their anxiolytic influences [5]. Lavender EO taken orally acts as low-dose benzodiazepines for calming nervousness in anxiety conditions as well [6].

Soil heavy metals (HMs) contamination is a globally recognized concern for soil scientists and environmentalists. HMs pollution is considered a potential warning to the environment and human health, and it is recognized as one of the most severe risks to the sustainability of agricultural ecosystems [7]. Some HMs play an essential dual role in plant metabolism. Several are crucial micronutrients; however, the same metals may become toxic when their concentration is exceeded a given level [8]. Throughout the recent few decades, increased human activities, rapid industrialization, and extended and over-developed agricultural practices have led to increased HMs pollutions worldwide. HMs are non-degradable, and their excessive accumulation in the soil affects the food chains, a significant concern for human health safety [9]. Many human activities, such as agricultural and industrial developments, transportation, coal combustion residues, petrochemical spills, and waste disposal, have caused the accumulation of HMs in agricultural soils [10].

Nickel (Ni) as a necessary element has a crucial function in plant metabolism and is necessary for the suitable function of diverse enzymes such as superoxide dismutase, urease, and glutathione and also takes roles in the detoxification of reactive oxygen species (ROS) in plants [11]. However, excessive Ni concentrations are toxic for most plant species and affect many physiological processes [12]. The tendency of Ni to compete with other cations such as Ca^2+^, Fe^2+^, and Zn^2+^ may cause their deficiency. Leather tanning, paint making, aluminum anodizing, steel production, electroplating, and cooling towers are the dominant sources of Ni pollution in soil and water and, are the main hazard to the sustainability of the environment, ecosystem, and food security [13]. The harmful concentrations of Ni obstruct the growth of plants and induce cell necrosis and black spots on the edge of leaves, reduce chlorophyll synthesis, retards the transfer of Fe to the leaves, and greatly decline the transpiration rate and leaf water potential [14].

Among the HMs, Pb is a toxic metal with harmful effects on the animal's biochemical processes, whose physiological mechanism is unknown yet [15]. In addition, Pb has been defined as an air and agricultural soils pollutant which severely reduces crop yields [16]. Due to the high accumulation of this metal on the soil surface, Pb is easily accessible to the plants, and over-absorption disrupts several biochemical pathways and the growth and productivity of plants [17]. In addition, Pb drastically impacts cell division, leads to leaves’ chlorosis, stops root and stem growth, reduces photosynthesis and DNA synthesis, and has destructive effects on many enzyme activities [18]. The decrease in plant functional parameters as a result of Pb toxicity has several reasons, such as the inhibitory effect of Pb on photosynthesis, reduction of CO_2_ fixation capacity, increase in metabolic costs of plants against the HMs stress, disruption of ion balance and a massive decrease of leaf area [19].

Exposure to HMs stress by inducing the over-production of ROS imposes several changes in plant metabolism. Generally, hydroxyl ion (OH^−^), hydrogen peroxide (H_2_O_2_), and superoxide (O_2_^−^) are the noxious radical species produced in different cell organelles [20]. The growth and productivity of the plant are influenced by ROS damage via oxidizing proteins, lipids, and nucleic acids [2]. Reactive oxygen species are commonly generated through plant metabolic processes which function as signaling molecules [20]. However, they are over-generated in higher amounts under stress conditions, causing oxidative stress, cell organelles injury, and even cell fatality [21]. Plants are strengthened by a set of physiological and molecular processes to withstand the abiotic stressors effect; among them, the antioxidant enzymes such as; ascorbate peroxidase (APX), superoxide dismutase (SOD), catalase (CAT), and guaiacol peroxidase (GPX), etc., play an essential function in stress tolerance. The excessive ROS accumulation triggers the expression of several genes to efficiently detoxify the stressors’ side effects [22].

Therefore, there is a crucial need for developing efficient strategies to improve tolerance mechanisms and enhance crop productivity in altering environments. The main approaches comprise mineral fertilizer applications or using suitable microorganisms which boost the tolerance mechanisms. Therefore, several biological methods, such as arbuscular mycorrhiza fungi (AMF) inoculation have been recommended to reduce the consequences of soil contamination with HMs [23]. Mycorrhiza fungi are beneficial microorganisms, their inoculation establishes symbiotic relationships with approximately 90% of plant species, containing bryophytes, ferns, and flowering plants [24], Phyto-stabilization of polyphosphate compounds, and HMs preservation by AMF mycelium enhance tolerance of plants to biotic stresses by forming the specialized radical mycelium [25]. Mycorrhiza fungi are obligate plant symbionts that colonize the plant roots via producing arbuscules and vesicles for exchanging immovable nutrients in favor of plant normal growth and development under stressful environments [26]. Extraradical mycelium development is a crucial constituent of the AMF-plant relationship. It connects colonized roots to the soil medium, where elements for example P are absorbed and transported to the underground and aerial parts of the plant [27]. In the host plant, the nutrients and water uptake were significantly enhanced by using AMF fungi. The host plant provides carbohydrates for the growth of the fungi. In return, the symbiotic behavior increases the photosynthesis rate by modulating the host plants' physiology and enhancing the P content and leaf surface area [28]. Moreover, AMFs are involved in the remediation of HMs by inducing a wide range of changes in the root absorption potential of the host plant. It was shown that AMF affect the bioavailability of HMs in the root medium of host plants, traps the HMs in the root system, and decreases their transfer to the aerial parts of the plant [29]. *Funneliformis mosseae* inoculation increased the phytoremediation of soils polluted by lead and nickel and also mitigated the growth potential and the yield of essential oil in lavender plants under HMs toxicity [30].

The present study aimed to assay the capability of AMF inoculation to counteract Pb and Ni stress in *Lavandula angustifolia* L., by evaluating the enzymatic and non-enzymatic antioxidant potential, and to estimate if AMF inoculation might promote the HMs phytoremediation and boost the plants' biochemical responses. Reportedly, this is the first study on the response of lavender plant’s enzymatic and non-enzymatic antioxidant pool to the HMs pollution under AMF inoculation.

## Materials and methods

### Plant materials, growth conditions, and treatments

The greenhouse conditions were a temperature regime of 21 and 25 °C at night and day, 8- and 16-h photoperiod for night and day, and a relative humidity of 70–75%.

The seeds of a local variety of *Lavandula angustifolia* L. were provided by Pakan Bazr seed company, Isfahan, Iran, and they were sown in trays(4 × 4 cm in cell size) containing cocopeat. The 3 months transplants with a height of 5–6 cm were planted in plastic pots (5 L) loaded with loamy-sandy-clay soil brought together from the farm of the University of Maragheh, at 0–30 cm soil depth. The characteristics of the soil are shown in Table 1 [31–33]. This investigation was carried out as a factorial experiment based on a completely randomized design (CRD) in four repetitions. The first factor was two concentrations of lead and nickel comprising 150 and 225 mg kg^−1^ soil from lead (II) nitrate and 220 and 330 mg kg^−1^ soil from nickel (II) nitrate as Pb and Ni pollutions. The heavy metals concentrations were two and three times more than the authorized standard in the soil based on the recommendation of the Water and Soil Organization of Iran (https://www.doe.ir/portal/file/?977240/soil-standard-1.pdf). The second factor was AMF (*Funneliformis mosseae*) inoculation containing 5 g kg^−1^ of soil and without AMF. The control lavender plants were cultivated in conditions without AMF and HMs treatments.

**Table 1:** The physicochemical characteristics of the soil sample used in the present experiment

Soil texture class	Sand (%)	Silt (%)	Clay (%)	Organic matter (%)	EC (dS m^−1^)	pH	Potassium (mg kg^−1^)	Phosphorous (mg kg^−1^)	Nitrogen (%)	Lead (mg kg^−1^)	Nickel (mg kg^−1^)
Loam sandy clay	55	15.5	29.5	1.53	1.23	7.35	494.15	8.59	0.082	0.068	0.027

The HMs dissolved in deionized water were sprayed on the soil every other day for two weeks at room temperature and regularly mixed to ensure soil pollution uniformity. Then, three lavender seedlings were transplanted into a 5 L pot. *Funneliformis mosseae* was provided by the Biology Laboratory of the Soil and Water Research Institute, Karaj, Iran. During the transplanting, *Funneliformis mosseae* inoculated soil was added under the root of lavender seedlings. All the pots were regularly watered with tap water throughout the experiment period. At 50% flowering stage, plant aerial parts were harvested. Some of the harvested materials were immediately frozen in liquid nitrogen and stored at -20 and -80 °C until the measurements. Moreover, another part of the aerial parts was air-dried under shade conditions for the essential oil component analysis.

### Root colonization

The roots of lavender plants were collected from the pots and rinsed with water to clean up the residual soil. The small pieces of fresh roots (1 cm) were cleansed in hot KOH (10%, w/v) for 10 min. The small fragments were washed out with deionized water and acidified by HCl (2%, v/v) for 15 min and smeared through trypan blue (0.05%) in lactic acid (80%, v/v) for 12 h (Phillips; Koske). Lastly, the stained roots were washed with deionized water and stored in a mixture comprising deionized water, glycerol, and lactic acid (1:1:1, v/v/v). The Olympus microscope (Olympus BH-2) distinguished the stained pieces. The blue fungus organs and hyphae were documented as photos. The colonization percentage was calculated by the protocol following Giovannetti and Mosse [34] as follows:

$$Percent\;colonization=\frac{Total\;infected\;roots}{Total\;noninfected\;roots}\times100$$

### Heavy metals content of the soil

Heavy metals (Pb and Ni) in the soil samples were measured during two experimental stages (before transplanting and after plant harvest). In this way, after drying the samples at room temperature, they were crushed and passed through a 2 mm sieve. In the next step, the samples were grounded to make the particles finer. Finally, 1 g of the soil was weighed from each treatment, and 10 ml of nitric acid and hydrochloric acid mixture (3:1 ratio) was added. After storage for 24 h, the samples were put down on the stove at 200 °C, and soil digestion was carried on up to the colors were removed and brown vapors appeared. After cooling, the samples were clarified using Whatman filter paper and volumized in 50 ml balloons. After preparing the samples, the Pb and Ni content was recorded by atomic absorption spectrometry (Shimadzu-AA6300-Kyoto, Japan) [35].

### Plant enzimatyc activities

For the determination of enzyme activity, 0.5 g of leaf fresh sample was extracted using potassium phosphate buffer (PPB) (pH = 6.9, 100 mM) comprising 4 mM Ethylenediaminetetraacetic acid and 1% Polyvinylpyrrolidone. The homogenate was centrifuged at 16,800 × g for 15 min. Finally, the supernatant was used for enzyme activity assay.

The activity of APX was estimated by Chen and Asada protocol (Chen and Asada, 1989). The reactance admixture contained 200 μl of ascorbate (2 mM), 750 μl of PPB (100 mM), 200 μl of hydrogen peroxide (2 mM), and 100 μl of the plant extract. The reaction was set off by the addition of H_2_O_2_, and the APX activity was measured using a spectrophotometer (Shimadzu model, UV 1800 model, Kyoto, Japan) by reducing the adsorption at 290 nm. APX activity was recorded as µmol ml^−1^ min^−1^ mg^−1^ protein with an extinction coefficient of 18.2 mM^−1^ cm^−1^.

The activity of GPX enzyme was assessed by Cakmak and Horst method (1991). The reaction mixture included 750 μl (100 mM) of PPB (pH = 7), 100 μl H_2_O_2_ (70 mM), 750 μl of guaiacol (10 mM), and 50 μl of the leaves extract. GPX enzyme activity was measured at 470 nm. The activity was displayed as µmol ml^−1^ min^−1^ mg^−1^ protein using the extinction coefficient of 16.26 mM^−1^ cm^−1^.

The SOD activity was recorded according to the protocol of Beauchamp and Fridovich [36]. The reaction mixture was composed of 1500 µl ‌of PPB (100 mM), 200 µL methionine (0.2 M), 100 µl EDTA (3 mM), ‌ 900 µl deionized water, and 100 µl sodium carbonate (Na_2_CO_3_) (1.5 M).‌ 100 µl of riboflavin and 50 µl ‌ of supernatant ‌ were combined. The combination was kept in complete darkness for 15 min. The optical density was read at 560 nm. SOD activity was expressed as µmol ml^−1^ min^−1^ mg^−1^ protein.

The Nguyen et al. procedure was employed to determine the polyphenol oxidase (PPO) activity [37]. 1 g of fresh plant tissue was squashed through 10 ml of PBB solution (pH = 6.7; 50 mM) embodying 10% polyvinylpyrrolidone and 0.5 M KCl, then it was separated by centrifugation at 32,928 × g for 15 min at 4 °C. The reaction combination embodied 1800 μl of PPB and 500 μl of pyrocatechol solution (0.5 M) and 700 μl of the separated solution. The optical density was evaluated at 420 nm for 120 s. PPO enzyme activity was recorded as µmol ml^−1^ min^−1^ mg^−1^ protein.

### Measurement of total antioxidant activity by the ferric-reducing ability of plasma (FRAP)

The method of FRAP was performed following the technique of Benzie et al. [38]. Fresh leaf (1 g) was homogenated using 2 ml of methanol and then centrifuged at 24,192 × g for 15 min. The assay uses 0.1 M acetate buffer solution (pH = 3.6) and 10 mM TPTZ [2, 4, 6-tris (2-pyridyl)—1, 3, 5-triazine]. 200 μl of the lavender plant extract was combined with 2950 µl of FRAP reagent and kept for 30 min. Lastly, the optical density was read at 593 nm using a spectrophotometer.

The DPPH (2,2-diphenyl-1-picryl-hydrazine-hydrate) protocol was also used to determine the total antioxidant capacity. 1 g of fresh leaves sample was extracted thru 2 ml of 80% methanol. Next, it was centrifuged at 32,928 × g for 15 min. 100 μl of the prepared extract was combined with 1900 μl of DPPH and placed in darkness for 30 min. The spectrophotometer read the absorption rate at 517 nm [39].

### Determination of Malondialdehyde (MDA) content

The content of MDA as a lipid peroxidation index was estimated by Heath and Packer protocol [40]. 0.5 g of plant fresh material was grounded with 1.5 ml TCA (0.1%) on ice and later centrifuged at 16,800 × g for 10 min at 4 °C. 500 µl of the separated homogenate was added to up to 1 ml of thiobarbituric acid (0.5%) solution and placed at room temperature for 95 min. The absorption was measured at three wavelengths of 440, 532, and 600 nm. Later, the MDA content was stated as nmol mg^−1^ fresh weight using the extinction coefficient of 155 mM^−1^ cm^−1^.

### Proline content estimation

The plant material (0.5 g) was grounded in sulfosalicylic acid (3%) solution to obtain a homogeneous extract and centrifuged at 16,800 × g for 25 min. The reaction combination was composed of 2 ml of the extract, ninhydrin, and glacial acetic acid and incubated for one hour at 100 °C, instantly laid on an ice pool to complete the reactance, and then 4 ml of toluene was added. The optical density was recorded at 520 nm [41].

### Measurement of total phenolics content (TPC), total flavonoids content (TFC), and anthocyanins content

To obtain TPC, a combination embodied of Folin-Ciocalteu substance (2500 µl), deionized water (450 μl), and methanolic extract (50 μl) was prepared and kept for 10 min. Then, 2 ml of Na_2_CO_3_·7H_2_O (7.5%) was added up and placed in the darkness for 2 more hours. Absorption was assessed at 765 nm with a spectrophotometer, and TPC was exhibited as mg gallic acid per 100 g^−1^ FW [42].

To quantify the TFC of lavender leaves, 1 g of fresh leaves was extracted with 4 ml of 96% ethanol. 500 μl of quercetin extract was added to 100 μl C_2_H_3_KO_2_ (1 M), 100 µl of aluminum chloride (10%), 1500 μl of methanol, and 2800 μl of deionized water. The mixture was kept at 25 °C for 30 min. The optical density of the mix was documented at 510 nm, and the TFC was documented as mg quercetin per 100 g^−1^ FW [43].

Total anthocyanin content was measured based on pH differences [44]. Lavender fresh leaves were placed in acidic methanol (2% HCl) for 24 h in darkness at 23–25 °C. The extract was diluted to the appropriate concentration with potassium chloride buffer (pH = 0.1). Two types of admixtures including one with KCl buffer (pH = 0.1) and the other with NaOAc buffer (pH = 4.5) were prepared. Absorption was assessed at 520 and 700 nm using a spectrophotometer. The total anthocyanin content was expressed in terms of 100 mg^−1^FW.

### Measurement of hydrogen peroxide (H_2_O_2_) content

To quantify the content of H_2_O_2_, 0.5 g of the fresh lavender leaf tissue was squashed with 1000 µl of trichloroacetic acid (0.1%), and the homogeneous mixture was centrifuged at 4 °C at 24,192 × g for 15 min. After that, 700 μl of zinc solution was combined with 700 μl of C_2_H_3_KO_2_ buffer (pH = 7, 10 μM) and 700 μl of KI (1 M). The optical density was assessed at 390 nm. The H_2_O_2_ content was plotted using a standard hydrogen peroxide curve and stated in µmol g^−1^ FW [45].

### GC–MS and GC-FID Analysis

Essential oils constituents were analyzed using GC–FID and GC–MS. The GC–MS analysis was done by an Agilent 7990 B gas chromatograph equipped with a 5988A mass spectrometer and an HP-5MS column (0.25 mm i.d., 30 m, 0.25 μm f.t., 5% phenyl methylpolysiloxane). The oven temperature programming was: 5 min at 60 °C, reaching 240 °C at 3 °C/min ramp, held for 10 min at the temperature. The helium (carrier gas) flow rate was 1 mL/min; the injector split ratio was 1:30; and the mass range and electron impact (EI) were 40–400 m/z and 70 eV, respectively. The oil constituents were identified by Adams [46] according to an interactive combination of linear retention indices (RIs), calculated against a homologous series of n-alkanes (C8–C40, Supelco, Bellefonte, CA, USA) and mass spectrum (MS) matching with libraries (ADAMS, WILEY 275 and NIST 17). The GC–FID analysis was carried out by an Agilent 7990 B gas chromatography connected to a flame ionization detector (FID), capillary column VF 5MS (30 m, 0.25 mm i.d., 0.50 μm f.t., 5% phenyl methylpolysiloxane). The above-mentioned oven temperature programming was employed. The injection volume was 1 μl of 1:100 (oil: hexane). The quantification of the oil components was carried out by considering the peak area normalization without correction factors [46].

### Statistical analysis

For two-way ANOVA analysis, MSTAT-C ver. 2.1 was used and the least significant difference test (LSD) was employed to compare the evaluated traits mean at 1% and 5% probability levels. The graphs were portrayed with Excel software. Heat map cluster and Pearson correlation analysis were illustrated using R studio ver. 14.2.1 software.

## Results

### Colonization by AMF inoculation

**Fig. 1:**
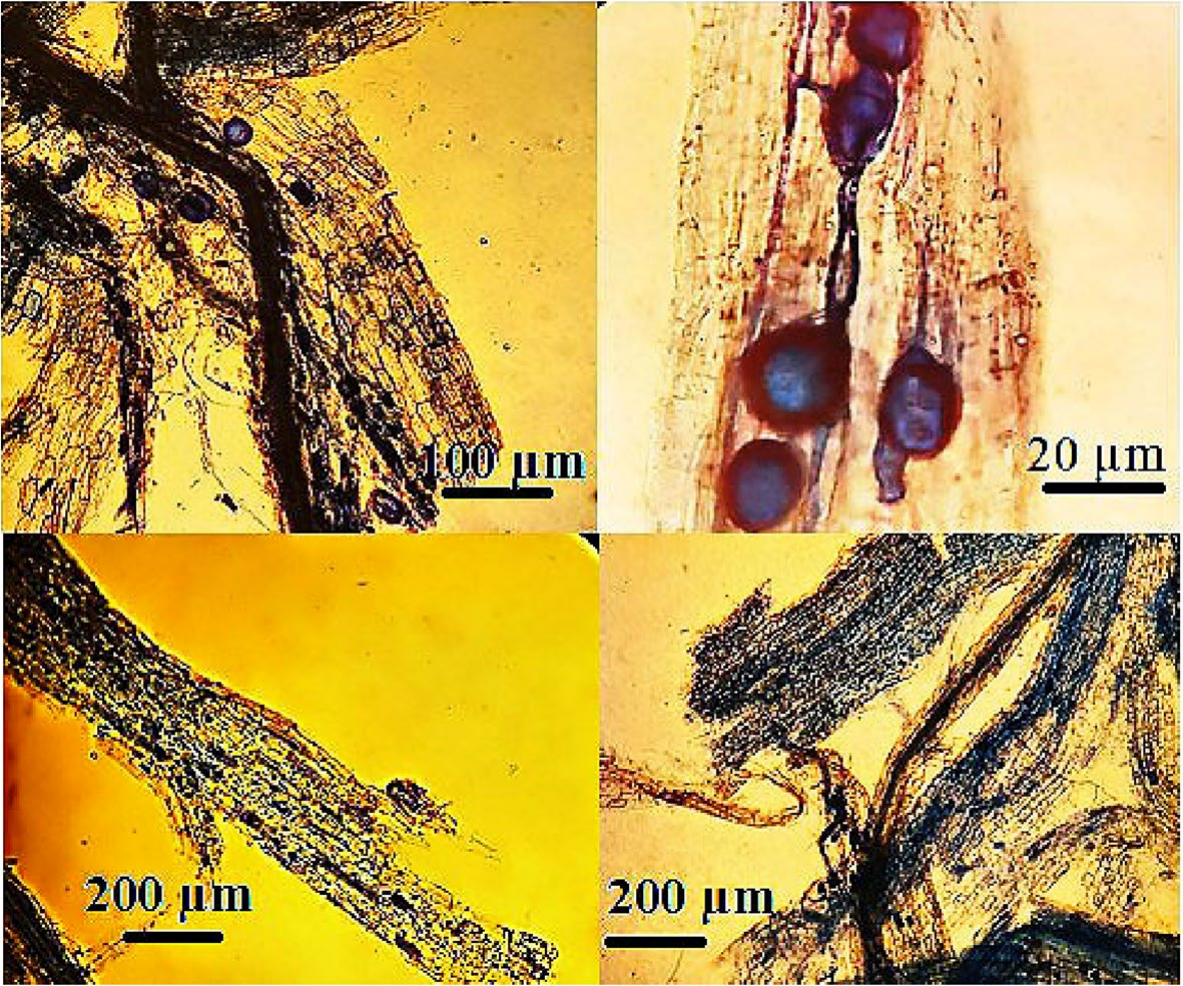
Microscopic images of the blotted pieces of lavender roots to distinguish colonization of *Funneliformis mosseae*

The microscopic high-quality photographs authenticated a reliable AMF colonization. The structures that emerged in the following pictures in blue color are *Funneliformis mosseae* organs and hyphae (Fig. 1).

### Shoot and root HMs content

**Fig. 2:**
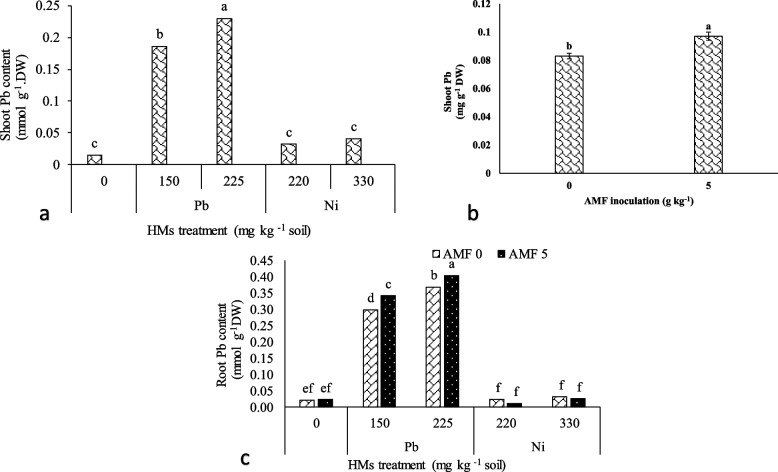
The influence of Pb and Ni stress on the Pb content of shoot (**a**) and root (**b**) in lavender plants treated with arbuscular mycorrhizal fungi (AMF). Dissimilar letters present significant differences based on the LSD test at 5% probability

The results revealed that the Pb content of shoots was influenced by Pb pollution and AMF usage (Table 2). The highest Pb content of the shoots was obtained at the maximum Pb pollution, which was 15.33% higher than the lavender plants without any pollution (Fig. 2a). Also, AMF treatment enhanced the shoots' Pb content by 16% over the plants without HMs treatment (Fig. 2b).

**Table 2:** The analysis of variance for the impact of arbuscular mycorrhiza fungi (AMF) treatment on the Pb and Ni content of the lavender plant under heavy metals (Pb and Ni) pollution

Source of variation	df	Shoot Pb	Shoot Ni	Root Pb	Root Ni
HMs	4	0.095**	0.011**	0.276**	0.044**
AMF	1	0.003**	0.004**	0.003**	0.001**
HMs + AMF	4	0.001 ns	0.002**	0.001*	0.001**
Error	30	0.001	0.001	0.001	0.001
Coefficient of variation		33.13	23.76	9.98	11.39

AMF and HMs refer to arbuscular mycorrhiza fungi and heavy metals (Pb and Ni), respectively. *, **, and ns, point to significant difference at *P* ≤ 5% and *P* ≤ 1% and non-significant, respectively

Pb content of roots was significantly influenced by HMs × AMF inoculation (Table 2). The most higher Pb content of the roots was recorded at 225 mg kg^−1^ Pb + AMF, and the minimum data was recorded at the control and also in the plants treated by Ni with/without AMF (Fig. 2c).

**Fig. 3:**
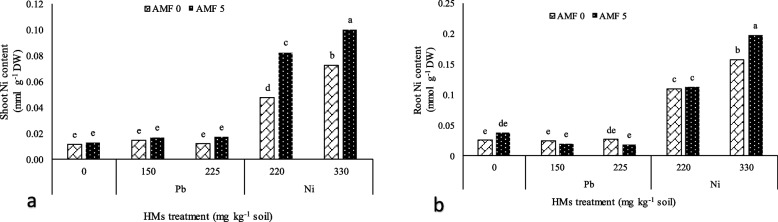
The influence of Pb and Ni stress on the Ni content of shoot (**a**) and root (**b**) of lavender plants treated with arbuscular mycorrhizal fungi (AMF). Dissimilar letters present significant differences based on the LSD test at 5% probability

Heavy metals × AMF inoculation significantly affected the shoots and roots Ni content (Table 3). The utmost shoots and roots Ni content was traced at 330 mg kg^−1^ Ni with AMF consumption and the lowermost was found at 225 mg kg^−1^ of Pb without AMF. The plants supplemented with 330 mg kg^−1^ of Ni + AMF had higher Ni uptake potential in the roots and shoots which was 900% and 690% over the control plants, respectively (Figs. 3a and b).

**Table 3:** AMF inoculation effects on the total antioxidant and enzyme activity of lavender plants under Pb and Ni heavy metals (HMs) stress

AMF (g kg^−1^ soil)	HMs (mg kg^−1^Soil)		FRAP (%)		DPPH (%)	APX (µmol ml^−1^ min^−1^)	GPX (µmol ml^−1^ min^−1^)	SOD (µmol ml^−1^ min^−1^)
**0**		0	23.06 h		3.181j	0.251e	0.6075f	11.77 h
	Pb	150	35.19e		17.93f	0.3225e	0.7e	32.68e
		225	41.5b		31.62b	0.615b	1.9125b	58b
	Ni	220	32.58f		14.01 h	0.285e	0.7125e	14.23 g
		330	35.72e		23.26d	0.4625 cd	1.2575c	14.79 g
**5**		0	30.98 g		7.765i	0.26e	0.6175f	28.68f
	Pb	150	38.03d		21.16e	0.36de	1.058d	54.67c
		225	44.29a		35.89a	0.91a	2.245a	61.02a
	Ni	220	36.21e		16.15 g	0.2925e	1.008e	31.75e
		330	40.3c		27.32c	0.5152bc	1.905b	38.51d
**LSD at 0.05%**			1.049		1.347	0.1119	0.3229	2.098
**S.O.V**			df					
**HMs**			4	267.921**	910.902**	0.111**	1.708**	2215.269**
**AMF**			1	180.073**	130.827**	0.835**	6.947**	2765.735**
**HMs** $\times$ **AMF**			4	9.189**	1.766*	0.068**	0.534**	132.506**
**Error**			30	0.697	0.636	0.006	0.050	2.111
**C.V**				4.80	4.03	18.18	23.53	4.20

AMF, HMs, S.O.V., and df were assigned to arbuscular mycorrhiza fungi and heavy metals (Pb and Ni), source of variation, and degree of freedom, respectively. * and **, significant at *P* ≤ 5% and *P* ≤ 1%, respectively. Dissimilar letters present significant differences based on the LSD test at 5% and 1% probability levels

### The antioxidant potential of plants

The results disclosed that the HMs pollution and AMF inoculation significantly affected FRAP and DPPH in lavender plants (Table 3). The highest levels of FRAP and DPPH were observed in the plants subjected to 225 mg kg^−1^ of Pb with AMF application. In contrast, the least antioxidant activity was recorded in the control plants without any treatment. So, the treatment with 225 mg kg^−1^ Pb + AMF showed an increase of up to 9.20 and 11.29 folds in the total antioxidant activity in both FRAP and DPPH methods compared to the control plants, respectively (Table 3).

The two-way ANOVA results revealed that the impact of HMs + AMF utilization was significant (P ≤ 0.01) on the APX enzyme activity in the lavender plant. The highest APX activity was related to 225 mg kg^−1^ Pb with AMF. While the lowest was obtained in the plants without any treatment. APX activity was boosted in 225 mg kg^−1^ Pb + AMF inoculation by 3.56 folds over to the control (Table 3).

The interaction effects of HMs and AMF were significant (*p* ≤ 0.01) on the activity of GPX enzyme in the lavender plants. The highest GPX activity was recorded in 225 mg kg^−1^ Pb with AMF, while, the lowest was obtained in the control plants. Alongside, GPX enzyme activity was increased by 3.69 folds under 225 mg kg^−1^ Pb + AMF application compared to the control (Table 3).

The co-application of AMF and HMs significantly (*p* ≤ 0.01) affected SOD activity. The highest activity of SOD was obtained in the plants treated with 225 mg kg^−1^ of Pb + AMF and, the lowest was obtained in the control plants. So, using 225 mg kg^−1^ Pb + AMF consumption increased SOD activity up to 418% over the control plants (Table 3).

**Fig. 4:**
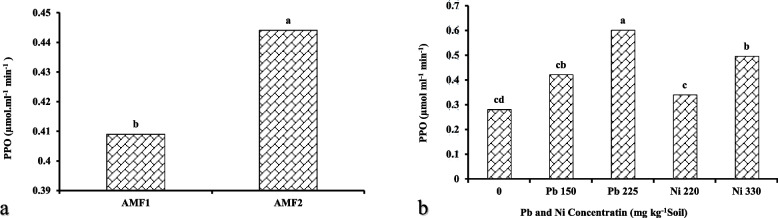
Mean comparisons for the effect of AMF (**a**) and Pb and Ni (**b**) on polyphenol oxidase (PPO) enzyme activity. AMF1 and AMF2 represent no mycorrhiza and application of mycorrhiza, respectively. Dissimilar letters present significant differences based on the LSD test at 5% probability

The application of AMF under the HMs stress had a significant impact (*p* ≤ 0.01) on the PPO activity in the lavender plant. PPO activity in the AMF-inoculated plants was 8.55% upper than in non-inoculated ones (Fig. 4a). Also, the maximum and the minimum activity of the PPO enzyme were recorded in the plants supplemented with 225 mg kg^−1^ of Pb and in the control plants, respectively. The PPO activity under the highest Pb treatment was 7.65 folds more than the control plants (Fig. 4b).

#### MDA content

HMs and AMF treatments influenced the MDA content of plants. The highest MDA content was recorded in 225 mg kg^−1^ of Pb + AMF. Contrarily, the lowest was observed in the control treatment. 225 mg kg^−1^ Pb + AMF boosted MDA content up to 97% more than the control (Table 4).

**Table 4:** The effects of AMF inoculation on some biochemical traits of lavender plants under Pb and Ni heavy metals (HMs) stress

AMF (g.kg^−1^)	HMs (mg.kg^−1^)		MDA (mg g^−1^ FW)		Proline (mg g^−1^ FW)	TPC (mg g^−1^ FW)	TFC (mg g^−1^ FW)	Anthocyanin (mg g^−1^ FW)	H_2_O_2_ (µmol g^−1^ FW)
**0**		0	0.795f		9.91j	25.64 g	40.58i	21.03j	10.76f
	Pb	150	0.905e		20.13d	60.13e	54.04ef	48.49e	13.51de
		225	1.148c		28.07b	81.56c	86.12b	59.46d	14.08d
	Ni	220	1.075 cd		15.03e	48.16f	47.65gh	46.53e	12.38e
		330	1.415c		20.44d	67.42d	63.94d	63.22c	12.69e
**5**		0	1.025d		11.27f	29.42 g	44.97hi	36.9f	11.52f
	Pb	150	1.393b		26.72b	81.14c	58.79de	54.87ed	19.52b
		225	1.67a		30.6a	110.8a	103.5a	71.09b	23.11a
	Ni	220	1.263bc		22.55c	63.42de	51.03 fg	66.35bc	13.35de
		330	1.57ba		27.1b	99.81b	75.68c	85.08a	16.59c
**LSD at 0.05%**			0.1119		2.027	5.329	6.369	5.598	1.191
**S.O.V**			df						
**HMs**			4	0.382**	244.426**	5648.761**	3391.758**	1316.948**	95.762**
**AMF**			1	2.048**	566.934**	4137.766**	692.224**	4251.019**	155.118**
**HMs** $\times$ **AMF**			4	0.153**	25.099**	262.349**	72.962**	419.183**	64.975**
**Error**			30	0.006	1.970	19.452	13.615	15.028	0.680
**C.V**				6.21	6.41	6.61	5.89	7.02	5.48

Dissimilar letters present significant differences based on the LSD test at 1% probability

^**^indicates a significant difference at a 1% probability level. S.O.V. and df refer to the source of variation and degree of freedom

#### Proline content

The AMF + HMs co-treatment meaningfully influenced the content of proline (*p* ≤ 0.01). The uppermost and the lowermost proline contents were recorded in the Pb treatment of 225 mg kg^−1^ + AMF and, the control plants. 225 mg kg^−1^ Pb + AMF raised proline content by 209% compared to the control (Table 4).

#### TPC, TFC, and anthocyanins content

The inoculation of AMF under Pb and Ni pollution significantly (*p* ≤ 0.01) influenced the TPC and TFC. 225 mg kg^−1^ of Pb + AMF inoculation enhanced the TPC and TFC up to 4.32 and 2.55 folds more than the control plants, respectively (Table 4).

The co-application of AMF, Pb, and Ni significantly affected anthocyanin content in the lavender plants. The highest anthocyanins content was obtained in Ni treatment of 330 mg kg^−1^ + AMF, and the lowest was obtained in the control plants. Moreover, 330 mg kg^−1^ Ni + AMF led to 4.07 folds increase in anthocyanins content over the control (Table 4).

#### H_2_O_2_ content

The two-way ANOVA findings revealed that Pb and Ni application + AMF inoculation impacted the content of H_2_O_2_. The content was the highest at the co-application of 225 mg kg^−1^ Pb + AMF. So, the mentioned treatment combination increased H_2_O_2_ content by 2.14 folds over to the control (Table 4).

#### Essential oil (EO) constituents

The chemical constituents of EO from lavender plants were examined by GC-FID and GC–MS analysis. As exhibited in Table 5, 26 constituents were recognized, comprising up to 83.64–95.09% of the total EO. The main constituents were borneol (15.81–28.91%), 1,8-cineole (5.03–12.75%), linalool (5.6–10.04%), linalyl acetate (6.68–10.36%), camphor (4.22–7.6%), caryophyllene oxide (3.26–7.15%), α-cardinol (1.54–4.02%), β-pinene (2.44–3.36%), santalene (1.50–2.88%), p-cymene (0.34–1.36%), α-amorphene (1.28–2.78%) and δ-3-carene (1.3–2.88%) (Table 5). The highest (28.91%) and the lowest (15.81%) percentages of borneol were recorded in the plants under AMF + 150 mg kg^−1^ of Pb and the control treatment without AMF application, respectively. Moreover, the uppermost 1,8-cineole (12.75%) content was obtained in AMF_-_treated plants (Table 5).

**Table 5:** Essential oil profile of lavender plants inoculated with AMF and exposed to Pb and Ni contamination

	Compounds	Retention index	AMF_1_	AMF_1_ + Pb 150	AMF_1_ + Pb 225	AMF_1_ + Ni 220	AMF_1_ + Ni 330	AMF_2_	AMF_2_ + Pb 150	AMF_2_ + Pb 225	AMF_2_ + Ni 220	AMF_2_ + Ni 330
1	α-Pinene	929.9	0.92 ± 0.23	0.76 ± 0.06	0.66 ± 0.24	0.89 ± 0.05	0.85 ± 0.25	1.01 ± 0.27	1.1 ± 0.31	0.93 ± 0.09	0.91 ± 0.06	0.62 ± 0.16
2	Camphene	944.1	1.17 ± 0.13	1.51 ± 0.06	0.84 ± 0.06	1.07 ± 0.18	1.1 ± 0.31	0.93 ± 0.08	1.01 ± 0.1	1.69 ± 0.1	0.64 ± 0.17	1.29 ± 0.23
3	Sabinene	969.2	1.91 ± 0.07	1.78 ± 0.11	1.15 ± 0.28	1.66 ± 0.11	2.35 ± 0.32	1.46 ± 0.11	1.63 ± 0.15	1.45 ± 0.22	1.01 ± 0.08	2.12 ± 0.45
**4**	***β*-Pinene**	**972**	**3.36 ± 0.16**	**2.44 ± 0.46**	**3.08 ± 0.14**	**3.06 ± 0.12**	**2.84 ± 0.29**	**2.86 ± 0.09**	**2.51 ± 0.24**	**3.19 ± 0.12**	**2.62 ± 0.23**	**2.79 ± 0.06**
5	*β*-myrcene	988.8	0.61 ± 0.09	2.31 ± 0.57	0.8 ± 0.12	0.88 ± 0.3	0.87 ± 0.36	1.06 ± 0.05	0.69 ± 0.06	2.34 ± 0.15	0.1 ± 0.05	0.89 ± 0.29
6	*n*-Decane	998.7	1.22 ± 0.23	1.12 ± 0.06	1.06 ± 0.06	1.24 ± 0.32	1.22 ± 0.12	1.02 ± 0.08	1.23 ± 0.10	1.18 ± 0.11	1.49 ± 0.15	0.93 ± 0.04
**7**	***δ*-3-Carene**	**1005.2**	**2.38 ± 0.39**	**1.3 ± 0.33**	**1.9 ± 0.15**	**2.38 ± 0.54**	**1.87 ± 0.18**	**2.17 ± 0.04**	**2.19 ± 0.18**	**2.13 ± 0.17**	**1.47 ± 0.28**	**2.04 ± 0.06**
**8**	**O-Cymene**	**1019.1**	**2.82 ± 0.05**	**3.25 ± 0.26**	**2.19 ± 0.31**	**2.6 ± 0.18**	**2.76 ± 0.15**	**2.77 ± 0.41**	**2.36 ± 0.12**	**3.01 ± 0.26**	**2.5 ± 0.13**	**2.44 ± 0.25**
**9**	**1.8-Cineole**	**1025.8**	**6.77 ± 1.96**	**7.94 ± 1.51**	**7.4 ± 2.07**	**5.03 ± 0.42**	**7.55 ± 0.84**	**12.75 ± 0.94**	**9.69 ± 1.25**	**9.97 ± 0.04**	**7.55 ± 0.31**	**8.78 ± 0.47**
10	(Z)-β-Ocimene	1033.7	0.77 ± 0.42	0.5 ± 0.04	0.31 ± 0.05	0.32 ± 0.19	-	0.69 ± 0.03	0.44 ± 0.17	0.54 ± 0.14	0.43 ± 0.21	0.26 ± 0.29
**11**	**Linalool**	**1098.3**	**8.54 ± 0.58**	**10.04 ± 2.67**	**9.91 ± 0.88**	**7.78 ± 0.33**	**7.63 ± 1.55**	**5.6 ± 0.37**	**3.94 ± 0.69**	**7.83 ± 0.99**	**15.1 ± 3.83**	**5.86 ± 0.14**
**12**	**Camphor**	**1139.6**	**5.03 ± 0.32**	**4.22 ± 0.38**	**6.25 ± 0.07**	**7.28 ± 1.05**	**5.25 ± 0.96**	**6.82 ± 0.31**	**7.6 ± 0.62**	**4.54 ± 0.1**	**6.21 ± 0.15**	**4.63 ± 0.5**
**13**	**Borneol**	**1160.3**	**15.81 ± 0.56**	**20.51 ± 1.44**	**20.22 ± 0.43**	**22.31 ± 1.8**	**22.64 ± 1.29**	**23.88 ± 0.23**	**28.69 ± 0.64**	**21.4 ± 1.48**	**23.36 ± 0.51**	**24.37 ± 1.24**
14	Terpinen-4-ol	1170.4	2.1 ± 0.66	1.12 ± 0.12	1.39 ± 0.2	1.31 ± 0.22	1.6 ± 0.65	0.5 ± 0.16	0.54 ± 0.12	1.45 ± 0.42	0.56 ± 0.1	0.54 ± 0.12
15	P-Cymene-8-ol	1185.6	1.15 ± 0.17	1.21 ± 0.1	1.06 ± 0.06	1.28 ± 0.19	0.51 ± 0.06	0.74 ± 0.04	0.9 ± 0.11	1.36 ± 0.04	0.34 ± 0.17	0.93 ± 0.05
16	Cryptone	1188.6	2.11 ± 0.15	2.17 ± 0.14	0.89 ± 0.5	1.99 ± 0.08	1.88 ± 0.11	1.54 ± 0.76	1.72 ± 0.07	2.13 ± 0.11	1.13 ± 0.2	2.41 ± 0.09
17	Bornyl formate	1219.8	1.54 ± 0.04	1.69 ± 0.06	1.53 ± 0.12	2.16 ± 0.24	1.43 ± 0.27	1.57 ± 0.29	1.81 ± 0.11	1.69 ± 0.08	1.3 ± 0.05	1.49 ± 0.05
**18**	**Linalyl acetate**	**1251.6**	**8.34 ± 2.27**	**6.77 ± 1.54**	**7.08 ± 1.23**	**7.7 ± 0.67**	**7.07 ± 1.73**	**6.68 ± 2.07**	**10.36 ± 1.5**	**8.69 ± 1.04**	**8.38 ± 1.14**	**7.26 ± 1.06**
19	Bornyl acetate	1284	1.53 ± 0.19	1.33 ± 0.13	0.78 ± 0.12	1.4 ± 0.19	1.18 ± 0.06	0.96 ± 0.44	1.07 ± 0.08	1.33 ± 0.05	0.65 ± 0.13	1.47 ± 0.04
20	Lavandulyl acetate	1286.8	3.17 ± 0.86	1.6 ± 0.21	1.78 ± 0.11	2.64 ± 0.43	1.59 ± 0.37	1.16 ± 0.26	1.65 ± 0.74	2.02 ± 0.11	1.32 ± 0.15	1.86 ± 0.81
21	Geranyl acetate	1380.6	0.32 ± 0.15	0.82 ± 0.15	0.71 ± 0.04	0.81 ± 0.04	0.3 ± 0.12	0.32 ± 0.04	0.25 ± 0.09	0.94 ± 0.06	0.34 ± 0.09	0.35 ± 0.05
**22**	**Santalene**	**1415**	**2.67 ± 0.58**	**2.72 ± 0.18**	**1.62 ± 0.04**	**2.5 ± 2.61**	**1.91 ± 0.32**	**2.66 ± 0.16**	**2.88 ± 0.43**	**1.98 ± 0.31**	**1.5 ± 0.18**	**2.68 ± 0.18**
23	Bergamotene	1431	1.13 ± 0.21	1.03 ± 0.06	1.08 ± 0.12	1.48 ± 2.16	0.92 ± 0.2	0.61 ± 0.14	0.44 ± 0.19	0.92 ± 0.05	0.38 ± 0.11	0.59 ± 0.06
**24**	α**-Amorphene**	**1482.7**	**1.69 ± 0.12**	**2.23 ± 0.33**	**2.48 ± 0.08**	**2.39 ± 1.74**	**2.37 ± 0.59**	**2.52 ± 0.30**	**2.72 ± 0.26**	**2.78 ± 0.06**	**1.28 ± 0.13**	**2.73 ± 0.78**
**25**	**Caryophyllene oxide**	**1580**	**3.7 ± 0.49**	**3.43 ± 0.16**	**5.82 ± 0.93**	**7.15 ± 3.06**	**3.81 ± 0.73**	**4.43 ± 0.17**	**4.18 ± 0.18**	**3.26 ± 1.06**	**3.88 ± 0.77**	**4.8 ± 0.65**
**26**	**α-Cadinol**	**1649**	**2.88 ± 0.56**	**3.99 ± 0.16**	**4.02 ± 0.04**	**3.62 ± 1.45**	**2.38 ± 0.44**	**3.57 ± 0.86**	**3.49 ± 0.26**	**2.35 ± 0.56**	**1.54 ± 0.22**	**3.66 ± 0.23**
	Total		83.64	87.79	86.01	92.93	83.886	90.28	95.09	91.1	86.005	87.79

## Discussion

The results confirmed the occurrence of mycorrhizal symbiosis at different levels of HMs (Fig. 1). The high HMs content in the AMF-inoculated plant tissues can be due to the extended networks of fungal hyphae and their subsequent penetration into the pores of the soil, which increase the absorption and transfer of heavy metals from the soil into the roots and other organs of the plant [47]. Therefore, the plants inoculated with AMF had the most phytoremediation potential. In agreement with our study, in research on onion and garlic, AMF coexistence resulted in a higher root colonization percentage [2].

With the high availability of Pb and Ni in the soil, the concentration of these HMs in the lavender plant increased (Figs. 3 and 4). In general, there are different mechanisms in the interaction of plants-AMF and the accumulation of HMs. The mechanisms are including; the accumulation in the apoplast, the establishment of ionic communications with cell wall components, the accumulation of phyto-chelates in the cytoplasm and later esterification, and finally, the accumulation of ions in vacuoles through metabolic processes [8]. On the other hand, AMFs create a direct connection between soil and the roots of the plant and improve the phytoremediation efficacy by modifying the availability of HMs, and concomitantly enhancing the plant tolerance [48]. Similar to our study, it has been reported that AMF colonization commonly improves the absorption and maintenance of HMs in roots and declines their transfer from roots to shoots. Therefore, AMF symbiosis intensifies the phytoremediation potential in soils contaminated with HMs [49].

Heavy metals toxicity stimulates the creation of ROS, which interrelate with some macromolecules such as DNA, proteins, and lipids, triggering a cascade of harmful processes collectively named “oxidative stress” [50]. Some previous experiments recommend that HMs adjust the cellular redox equilibrium in some imaginable ways by directly inhibiting the antioxidant enzymes' activities such as by substituting essential cations from the specific binding positions and targeting their -SH groups in SOD, APX, and GPX [51]. Chowardhara et al. (2020) reported enhanced enzymatic antioxidants activities containing APX, SOD, GPX, and CAT under HMs pollution in *B. juncea*, demonstrating an essential role of the enzymes in the HMs-stress tolerance [52]. Pan and co-workers revealed that the expression of various genes encoding for enzymatic antioxidants such as *LuSOD1*, *LuPOD1*, and *LuPOD2* increased in *Linum usitatissimum* L., which led to more tolerance to Pb-toxicity [53]. As already known, superoxide radical is the immediate response of the antioxidant system produced during stress which quickly converts the superoxide radical into hydrogen peroxide and molecular oxygen [54]. Based on these results, it can be stated that AMF coexistence affects plant metabolism by motivating the synthesis of secondary metabolites, and potentially by improving the accumulation of antioxidant compounds in plants. The idea is that the enhanced antioxidant potential in fungus-treated plants regulates oxidative reactions and antioxidant defense by increasing the activity of antioxidant enzymes, including APX, which scavenge the ROS radicals over-production under stress conditions [55]. The AMF treatment leads to alterations in the plant hormones concentration, which improves the uptake of nutrient elements and concomitantly leads to more antioxidant activity [56]. Also, Giri et al. [57] noted that AMF inoculation enhanced the antioxidant activities in the host plant, especially the PPO enzyme, and resulted in a reliable stress adjustment. Accordingly, under Pb stress, PPO activity stimulates the activation of ascorbate peroxidase (APX) from the cell wall [58]. Our results are consistent with the above-mentioned findings on the response of lavender plants to AMF inoculation exposed to HMs stress.

Moreover, our findings showed that Pb and Ni toxicity increased MDA content, while it was reduced by AMF inoculation. The over-generation of MDA is the initial signal of membrane lipid peroxidation, resulting from the destruction of unsaturated fatty acids, and cell membrane breakage, which can be utilized for the quantification of the stress damage [59]. The increase in MDA production can be attributed to the strengthened peroxidation of unsaturated fatty acids, the attack of free radicals on lipids, and the production of various aldehydes [60]. On the other hand, AMF usually improves the growth of the host plant by reducing the toxicity of HMs. This phenomenon is closely related to the fortifying of the antioxidant defense, which enhances the activity of antioxidants [61]. As a result of HMs toxicity; the production of ROS increases, leading to the oxidation of cell membrane lipids, and then MDA content drastically increases (Khan et al., 2016). AMF treatment reasonably reduced ROS accumulation with an increment in the antioxidant enzyme activity [61], which is in line with the current study.

In the present experiment, by increasing the HMs levels + AMF inoculation, the proline content was increased. Proline enhancement in the AMF inoculated plants under HMs stress can be due to the more absorption of metal elements involved in proline biosynthesis [62]. Aghaei et al. [63] stated that under stress conditions, AMF treatment reduced protein synthesis, stimulated protein hydrolysis, and enhanced abscisic acid concentration leading to the accumulation of amino acids such as proline. As known, proline concentration is related to the production of soluble sugars. Therefore, with the increase in the production of soluble sugars due to higher nutrient absorption, the synthesis of glutamate as the precursor of proline is enhanced to overcome stress depression [64].

The enhanced biosynthesis of TPC and TFC was a response to the priority of AMF inoculation and HMs contamination in lavender plants. Phenolic compounds are described as an important biomarker of metal toxicity. The increased content of these compounds has been stated in several plant species [65]. These compounds have antioxidant activity in response to stressful conditions, conceivably owing to their electron-donating nature [66]. The accumulation of TPC and TFC in mycorrhizal treated plants can be a reason for the involvement of these compounds in modulating symbiosis behavior. Reversely, this verifies the stimulation of those compounds' production by the AMF inoculation [67]. Phenolic compounds keep roles in the AMF symbiosis process by affecting spore germination, growth of fungal hyphae, and stimulation of fungal cloning in roots. TFCs, as antioxidants and metal-chelating compounds, essentially mediate the tolerance to HMs stress in plants [68]. They are antioxidant components with functional hydroxyl and carboxyl groups to bind specifically with HMs and can eliminate ROS produced under HMs stress [69].

In our experiment, AMF inoculation under HMs stress increased the anthocyanin content of the lavender plants. The AMF treatment triggers the accumulation of anthocyanins in the host plant by modulating enzyme activities and by changing several physiological mechanisms involved in the antioxidant pool [70]. Anthocyanins play a role in the detoxification of HMs through the formation of metal-anthocyanin complexes and hence protect the plant against ROS with antioxidant properties [29]. The enhanced anthocyanin accumulation under the HMs polluted conditions is related to the over-expression of anthocyanin biosynthesizing genes, which helps to immobilize metal ions and subsequently increase the tolerance of plants [71]. The increased levels of anthocyanins in the AMF-inoculated plants owing to the improved nutritional conditions more seemingly enhance the biosynthesis of diverse secondary metabolites [72]. In agreement with our findings, the highest anthocyanin content was reported in *F. mosseae*-inoculated eggplants [73].

Our results showed that H_2_O_2_ content increased in plants inoculated with AMF under HMs stress. Shahabivand et al. [74] reported that the content of H_2_O_2_ was higher in sunflower plants under Cd stress + AMF than in control plants. At low concentrations, H_2_O_2_ is detoxified by the plant's antioxidant system and even acts as a signaling molecule in the defense systems of plants against stress [59]. The increase in the activity of antioxidant enzymes such as the case in our study indicates that the accumulation of H_2_O_2_ led to the protection of the plant against the HMs stress [75]. Also, any increase in the MDA content under stress conditions verifies the peroxidation of membrane lipids due to free radicals, including H_2_O_2_ [76]. Many studies also reported H_2_O_2_ as a signaling molecule in plant responses to diverse biotic and abiotic stressors [77]. Similar to our results, H_2_O_2_ accumulation was detected in *S. cannabina* plants under saline stress after AMF colonization which is a shred of evidence that H_2_O_2_ controls the induction of strigolactone biosynthesis by the AMF inoculation via mitigating the stress conditions [78].

The current findings revealed that the lavender EO content was improved under mild HMs stress conditions. The EO biosynthesis under stress conditions is a primary protection mechanism in medicinal and aromatic plants [79]. Also, the photosynthetic rate reduces owing to the lessening in CO_2_ uptake consequently shutdown stomata, leading to the production of NADPH^+^ [80]. The improvements in the content of the EO constituents are indicators of the limited plant productivity under stress conditions in medicinal and aromatic plants [81]. It is more evident that the AMF treatment enhances nutrient availability and promotes metabolic pathways (e.g., nutrient transport), which has a vital role in the essential oil production in glandular trichomes, EO channels, and secretory ducts [82]. Also, AMF aid to change inorganic N into organic N in the proteins and chlorophyll structures, which causes an enhancement in the photosynthetic rate [83]. It has been reported that improvements in principal photosynthetic compounds content such as erythrose‐4‐phosphate, phosphoenol pyruvate, pyruvate, and glyceraldehyde‐3‐phosphate which play the primary role in the biosynthesis of terpene components and the accumulation of essential oils in medicinal and aromatic plants [84]. Additionally, Hazzoumi et al. [85] reported that AMF application induced EO accumulation by increasing the endogenic hormone levels, mainly cytokinins, indole‐3‐acetic acid, and gibberellins. In the same way, Hazzoumi et al. [85] showed that the EO components of *Ocimum basilicum* were increased under stress conditions.

## Conclusion

The findings revealed that *Funneliformis mosseae* improved the phytoremediation potential of Pb and Ni, and also drastically enhanced the antioxidant enzymes activity, MDA, and proline content in lavender plants. Moreover, the AMF inoculation under mild HMs stress conditions enhanced the content of the main components of lavender essential oils such as borneol, 1,8-cineol, camphor, linalool, and linalyl acetate, while their content was reduced at higher HMs toxicity levels, especially under higher Pb pollution levels. Our findings could be extremely appealing in the agricultural and environmental sections, especially in the soils contaminated with mild levels of HMs. These marginal land are commonly not suitable for the production of common crops. However, medicinal plants could be a suitable candidate for exploitation in these low-grade lands. And since HMs are mainly aggregate in the vegetative organs of plants, and the essential oils are free from HMs; as a result, we can conclude that *Lavandula angustifolia* inoculated with AMF is enough efficient for phytoremediation of Pb and Ni polluted soils while keeping its normal growth and essential oil production. Considering the fact that AMF inoculation induced more tolerance responses in the polluted soils; the recommendation is that in the future, try to comparatively study several AMF types and even other microorganisms such as bacteria to reach a more efficient tolerance and phytoremediation protocol in the polluted soils.

## Data Availability

The datasets used and/or analyzed during the current study are available from the corresponding author upon reasonable request.

## Abbreviations

HMs: Heavy metals
AMF: Arbuscular mycorrhiza fungus
MDA: Malondialdehyde
TPC: Total phenolic content
TFC: Total flavonoid content
EO: Essential oil
DPPH: 2,2-Diphenyl-1-picrylhydrazyl
FRAP: Ferric reducing antioxidant power
Pb: Lead
Ni: Nickel
ROS: Reactive oxygen species
ANOVA: Analysis of variance
APX: Ascorbate peroxidase
CAT: Catalase
SOD: Superoxide dismutase
GPX: Guaiacol peroxidase
PPB: Potassium phosphate buffer
EDTA: Ethylenediaminetetraacetic acid
TPTZ: 2,4,6-Tri(2-pyridyl)-s-triazine
TCA: Trichloroacetic acid
GS-MS: Gas chromatography-mass spectrometry
FID: Flame ionization detector
EI: Electron impact
